# Soluble Extracts from Chia Seed (*Salvia hispanica* L.) Affect Brush Border Membrane Functionality, Morphology and Intestinal Bacterial Populations In Vivo (*Gallus gallus*)

**DOI:** 10.3390/nu11102457

**Published:** 2019-10-14

**Authors:** Bárbara Pereira da Silva, Nikolai Kolba, Hércia Stampini Duarte Martino, Jonathan Hart, Elad Tako

**Affiliations:** 1 Department of Nutrition and Health, Federal University of Viçosa, Viçosa 36570000, Minas Gerais, Brazil; barbarapereira2805@gmail.com (B.P.d.S.); hercia72@gmail.com (H.S.D.M.); 2 USDA-ARS, Robert W. Holley Center for Agriculture and Health, Cornell University, Ithaca, NY 14853, USA; nikolai.kolba@ars.usda.gov (N.K.); jjh16@cornell.edu (J.H.)

**Keywords:** intra amniotic (in ovo) administration, zinc gene expression, iron gene expression, brush border membrane functional genes, intestinal bacterial populations, villus surface area

## Abstract

This study assessed and compared the effects of the intra-amniotic administration of various concentrations of soluble extracts from chia seed (*Salvia hispanica* L.) on the Fe and Zn status, brush border membrane functionality, intestinal morphology, and intestinal bacterial populations, in vivo. The hypothesis was that chia seed soluble extracts will affect the intestinal morphology, functionality and intestinal bacterial populations. By using the *Gallus gallus* model and the intra-amniotic administration approach, seven treatment groups (non-injected, 18 Ω H_2_O, 40 mg/mL inulin, non-injected, 5 mg/mL, 10 mg/mL, 25 mg/mL and 50 mg/mL of chia seed soluble extracts) were utilized. At hatch, the cecum, duodenum, liver, pectoral muscle and blood samples were collected for assessment of the relative abundance of the gut microflora, relative expression of Fe- and Zn-related genes and brush border membrane functionality and morphology, relative expression of lipids-related genes, glycogen, and hemoglobin levels, respectively. This study demonstrated that the intra-amniotic administration of chia seed soluble extracts increased (*p* < 0.05) the villus surface area, villus length, villus width and the number of goblet cells. Further, we observed an increase (*p* < 0.05) in zinc transporter 1 (ZnT1) and duodenal cytochrome b (Dcytb) proteins gene expression. Our results suggest that the dietary consumption of chia seeds may improve intestinal health and functionality and may indirectly improve iron and zinc intestinal absorption.

## 1. Introduction

Micronutrient deficiency affects approximately two billion people worldwide. Iron (Fe) and zinc (Zn) deficiencies are the most prevalent, affecting approximately 45% and 17%, respectively, of the world population [1,2,3]. Both mineral deficiencies are more prevalent in Africa, South East Asia and Latin America [4,5]. Among the dietary factors that contribute to Fe and Zn deficiencies is their low bioavailability due to dietary potential inhibitors, such as phytic acid and phenolic compounds [2,6,7]. Dietary Fe and Zn deficiencies affect normal cell division and differentiation, as well as growth and development, impair physical and cognitive development, and increase the risk of infection [4,7,8].

We have previously established the *Gallus gallus* as a model to assess dietary Fe and Zn bioavailability [9,10,11,12,13,14,15]. In addition, this experimental model presents a complex gut microbiota [16], as the phylum level was shown to be similar to humans [17,18]. Further, the intra amniotic administration method has been widely used and demonstrates the potential prebiotic effects of soluble fibers from beans, chickpeas, lentil and wheat, with demonstrated effects on the intestinal functionality, morphology, and microbial populations [10,13,15]. 

Prebiotics are dietary substrates that selectively promote the proliferation and/or activity of health-promoting bacterial populations in the colon [19,20]. The soluble extracts are obtained by the isolation process of the prebiotics of the food matrix and are composed for the most part of soluble fiber. The most commonly used prebiotics, as inulin, raffinose and stachyose, are dietary fibers with a well-investigated and proven ability to promote the abundance of intestinal bacterial populations, which may provide additional health benefit to the host [21]. It is known that soluble extracts are responsible for improving gastrointestinal motility [22,23], intestinal functionality and intestinal morphology [10,13,24,25], and improving mineral absorption [10,26]. Recent Studies have shown that the consumption of plant seed origin soluble extracts can up regulate the gene expression of brush border membrane (BBM) proteins that contribute to the digestion and absorption of nutrients, such as sucrase-isomaltase, aminopeptidase and sodium glucose cotransporter-1 [10,11,13]. Further, soluble extracts can positively affect intestinal health by increasing mucus production, goblet cell number, goblet cell diameter, villus surface area, villus height, villus width, and crypt depth [10,13,15,27,28]. These functional and morphological effects appears to occur due to the increased motility of the digestive tract by the soluble extracts, leading to hyperplasia and/or hypertrophy of muscle cells [29]. In addition, plant origin soluble extract (with high fiber content and, therefore, potential prebiotic properties) administration may act, directly or indirectly, as a factor that increases iron and zinc bioavailability [30,31,32]. This event occurs due the lower intestine (colon) fiber fermentation process and the bacterial production of short-chain fatty acids (SCFAs) that reduce the intestinal pH, inhibiting the growth of potentially pathogenic bacterial populations and increasing the solubility and, therefore, the absorption of minerals [10,26]. The SCFAs can increase the proliferation of epithelial cells, which, in return, increases the absorptive surface area, which contributes to the absorption of dietary minerals [33]. Also, it was previously shown that the consumption of soluble extracts has a synergistic effect, as it promotes the metabolic interactions within the gastrointestinal microbial community via the production of organic acids, which provide an acidic environment in the colon, indirectly suppressing the growth of pathogens [34]. 

The use of iron- and zinc-rich foods may be a good strategy aimed to reduce the prevalence of iron and zinc deficiencies, respectively. Chia (*Salvia hispanica* L.) is an herbaceous plant with good nutritional and functional value with high concentrations of bioactive compounds such as dietary fiber and minerals, including iron and zinc [35]. Although iron and zinc are present in high concentrations, it is important to take into account the bioavailability of these minerals [36]. In the present study, chia was chosen as the soluble extract source, since the consumption of chia bacame extensively common worldwide, and specifically consumed with increasing amounts in Mexico, Argentina, Chile, New Zealand, Japan, USA, Canada and Australia [37], as in some of these geographical regions (e.g., South America), dietary Fe and Zn deficiencies are a major health concern [4,5]. Thus, the primary objective of this study was to assess the effects of the intra-amniotic administration of chia soluble extracts with a putative prebiotic effect on Fe and Zn status and brush border membrane functionality, in vivo. A secondary objective was to evaluate the effects of the tested extracts on intestinal bacterial populations. The third objective was to evaluate the effects of the chia soluble extracts on intestinal morphology. We hypothesized that the chia soluble extracts will affect the intestinal morphology, functionality and bacterial populations.

## 2. Material and Methods

### 2.1. Sample Preparation

Chia seeds (*Salvia hispanica* L.) grown in the state of Mato Grosso (Brazil) were used for this study. To obtain the flour, the seeds were ground up in three replicates, using a knife mill (Marconi Equipment, Algodoal, Brazil), to a particle size of 850 μm. Subsequently, chia flour was packed in polyethylene aluminum bags and stored in a freezer (−20 °C) until analysis.

### 2.2. Polyphenols Analysis

#### 2.2.1. Chia Sample Preparation

A volume of 5 mL of methanol:water (50:50 *v/v*) was added to 0.5 *g* of chia flour. The resulting slurry was vortexed for 1 min before incubation in a 24 °C sonication water bath for 20 min at room temperature. Samples were again vortexed and placed on a rocker at room temperature for 60 min before centrifuging at 4000× *g* for 15 min. Supernatants were filtered with a 0.2 μm PTFE syringe filter and stored at −20 °C for later use.

#### 2.2.2. Liquid Chromatography–Mass Spectrometry (LC-MS) Analysis

Extracts and standards were analyzed by an Agilent 1220 Infinity Liquid Chromatograph (LC; Agilent Technologies, Inc., Santa Clara, CA, USA) coupled to an Advion expressionL^®^ compact mass spectrometer (CMS; Advion Inc., Ithaca, NY, USA). Ten-microliter samples were injected and passed through an XBridge Shield RP18 3.5 µm 2.1 × 100 mm column (Waters, Milford, MA, USA) at 0.6 mL/min. The column was temperature-controlled at 40 °C. The mobile phase consisted of ultra-pure water with 0.1% formic acid (solvent A) and acetonitrile with 0.1% formic acid (solvent B). Polyphenols were eluted using linear gradients of 94.0 to 84.4% A in 1.50 min, 84.4 to 81.5% A in 2.25 min, 81.5 to 77.0% A in 6.25 min, 77.0 to 55.0% in 1.25 min, 55.0 to 46.0% in 2.25 min, 46.0 to 94.0% in 2.25 min and hold at 94.0% A for 2.25 min for a total run time of 18 min. From the column, the flow was directed into a variable wavelength Ultraviolet (UV) detector set at 280 nm. The flow was then directed into the source of an Advion expressionL^®^ CMS, and Electrospray ionization (ESI) mass spectrometry was performed in the negative ionization mode using selected ion monitoring with a scan time of 200 ms. The capillary temperature and voltages were 250 °C and 180 volts, respectively. The ESI source voltage and gas temperature were 2.5 kilovolts and 250 °C, respectively. The desolvation gas flow was 240 L/h. Advion Mass Express™ software (Advidon, Ithaca, USA) was used to control the LC and compact mass spectrometers (CMS) instrumentation and data acquisition. Individual polyphenols were identified and confirmed by comparison of *m/z* and LC retention times with authentic standards. The analysis of MS and UV data was performed using Advion Data Express™ software (Advidon, Ithaca, USA).

### 2.3. Extraction of Soluble Extracts from Chia

The extraction of prebiotics was performed according to Tako et al. [14], Hou et al. [13] and Pacific et al. [10]. Chia flour samples were dissolved in distilled water (50 g/L) (60 °C, 60 min) and centrifuged at 3000 rpm (4 °C) for 25 min, and then the supernatant was collected. The supernatant was then dialyzed (MWCO 12–14 kDa) (48 h) against distilled water. The dialysate was collected and lyophilized to yield a fine off-white powder [12].

### 2.4. Phytate, Dietary Fiber, Iron and Zinc Analysis in Chia Seeds and Chia Extract

Dietary phytic acid (phytate)/total phosphorous was measured as phosphorus released by phytase and alkaline phosphatase, according to manufacturer’s instructions (*n* = 5) (K-PHYT 12/12. Megazyme International, Bray, Ireland). The determination of total fiber and soluble and insoluble fractions was performed by the enzymatic-gravimetric method according to AOAC [38], using enzymatic hydrolysis for a heat-resistant amylase, protease and amyloglucosidase (Total dietary fiber assay Kiyonaga, Sigma^®^, Kawasaki, Japan). For the determination of iron and zinc, chia seed and chia extract (0.5 g) were treated with 3.0 mL of a 60:40 HNO_3_ and HClO_4_ mixture in a Pyrex glass tube and left overnight to destroy organic matter. The analyses were performed using an inductively coupled plasma atomic emission spectrometer (ICP-AES) (Thermo iCAP 6500 series, Thermo Jarrell Ash Corp., Franklin, MA, USA) [12,28].

### 2.5. Animals and Design 

Cornish-cross fertile broiler eggs (*n* = 105) were obtained from a commercial hatchery (Moyer’s chicks, Quakertown, PA, USA). The eggs were incubated under optimal conditions at the Cornell University Animal Science poultry farm incubator. All animal protocols were approved by the Cornell University Institutional Animal Care and Use committee (ethic approval code: 2007-0129). 

#### Intra Amniotic Administration

Lyophilized soluble extracts were diluted in 18 Ω H_2_O and for sample osmolarity determination (≤320 OSM). At 17 days of embryonic incubation, eggs containing viable embryos were weighed and divided into 7 groups (*n* = 15). All treatment groups were assigned eggs of a similar weight frequency distribution. Each group was then injected with the specified solution (1 mL per egg), using a 21 gauge needle into the amniotic fluid, which was identified by candling. The 7 groups were assigned as follows: (1) non-injected; (2) 18 Ω H_2_O; (3) inulin (40 mg/mL); (4) chia seed extract 0.5% (5 mg/mL); (5) chia seed extract 1% (10 mg/mL); (6) chia seed extract 2.5%; (7) chia seed extract 5% (50 mg/mL). After the injections, the holes were sealed with cellophane tape and the eggs were placed in hatching baskets. Immediately after hatch (21 days), the chicks were euthanized by CO_2_ exposure and their small intestine, blood, pectoral muscle, cecum and liver were collected.

### 2.6. Iron and Zinc Content in Serum and Liver

Liver (0.5 g) and serum (50 µL) were treated with 3.0 mL of a 60:40 HNO_3_ and HClO_4_ mixture in a Pyrex glass tube and were incubated overnight. The mixture was then heated to 120 °C for two hours and 0.25 mL of 40 µg/g Yttrium was added as an internal standard. Next, the temperature of the heating block was raised to 145 °C for 2 h. Then, for 10 min, the temperature of the heating block was raised to 190 °C. The cooled samples were then diluted to 20 mL, vortexed and transferred into autosampler tubes to be analyzed via inductively coupled plasma atomic emission spectrometer (ICP-AES). (Thermo Jarrell Ash Corp., Franklin, MA, USA) [12,28].

### 2.7. Isolation of Total RNA from Duodenum and Liver 

Total RNA was extracted from 30 mg of the proximal duodenal tissue or liver tissue (*n* = 10) using Qiagen RNeasy Mini Kit (RNeasy Mini Kit, Qiagen Inc., Valencia, CA, USA) according to the manufacturer’s protocol. Total RNA was eluted in 50 µL of RNase-free water. All steps were carried out under RNase-free conditions. RNA was quantified by absorbance at A 260/280 and the integrity of the 18S ribosomal RNAs was verified by 1.5% agarose gel electrophoresis followed by ethidium bromide staining. RNA was stored at −80 °C.

### 2.8. Real Time Polymerase Chain Reaction (RT-PCR)

To create the cDNA, a 20 µL reverse transcriptase (RT) reaction was completed in a BioRad C1000 touch thermocycler using the Improm-II Reverse Transcriptase Kit (Catalog #A1250; Promega, Madison, WI, USA). The concentration of cDNA obtained was determined by measuring the absorbance at 260 and 280 nm using an extinction coefficient of 33 (for single stranded DNA). Genomic DNA contamination was assessed by a real-time RT-PCR assay for the reference gene samples [12].

### 2.9. Primer Design

The primers used in the real-time qPCR were designed based on 13 gene sequences from the Genbank database, using Real-Time Primer Design Tool software (IDT DNA, Coralvilla, IA, USA). The sequences and the description of the primers used in this work are summarized in Table 1. The specificity of the primers was tested by performing a BLAST search against the genomic National Center for Biotechnology Information (NCBI) database. The *Gallus gallus* primer 18S rRNA was designed as a reference gene. Results obtained from the qPCR system were used to normalize those obtained from the specific systems as described below.

### 2.10. Real-Time qPCR Design

All procedures were conducted as previously described [10,11,12,13]. The specific primers that were used are shown in Table 1.

### 2.11. Collection of Microbial Samples and Intestinal Content DNA Isolation

The cecum contents were removed under sterile conditions, placed into a sterile tube containing 9 mL of Phosphate buffered saline (PBS) and homogenized by vortexing with glass beads for 3 min [27,39]. All procedures were conducted as previously described [10,11,12,13,14].

### 2.12. Primer Design and PCR Amplification of Bacterial 16S rDNA

Primers for *Lactobacillus*, *Bifidobacterium*, *Clostridium* and *Escherichia coli* were used [16,39]. The universal primers were designed with the invariant region in the 16S rRNA of bacteria and were used as internal standards. The proportions of each bacterial group are presented. The PCR products were loaded on 2% agarose gel stained with ethidium bromide and quantified by Quantity One 1-D analysis software (Bio-Rad, Hercules, CA, USA) [12]. The evaluation of the relative abundance of each examined bacterium was conducted as previously described [10,11,12,13,14].

### 2.13. Glycogen Analysis

At hatch, the pectoral muscle (20 mg) was collected for glycogen analysis. The tissue samples were homogenized in 8% perchloric acid, and glycogen concentration was determined as previously described [40]. After homogenization, the samples were centrifuged at 12,000 rpm at 4 °C for 15 min. The supernatant was removed, and 1.0 mL of petroleum ether was added. After mixing, the petroleum ether fraction was removed, and samples from the bottom layer were transferred to a new tube containing 300 µL of color reagent. All samples were read at a wavelength of 450 nm in an ELISA reader and the amount of glycogen was calculated according to a standard curve. The amount of glycogen present in pectoral sample was determined by multiplying the weight of the tissue by the amount of glycogen per 1 g of wet tissue. 

### 2.14. Morphological Examination 

As previously described [10,41], liver and intestine samples were collected at the conclusion of this study. Samples were fixed in 4% (*v/v*) buffered formaldehyde, dehydrated, cleared, and embedded in paraffin. Serial sections of 5 µm were obtained and were deparaffinized in xylene, rehydrated in a different concentration of alcohol, stained with hematoxylin/eosin or Alcian Blue/Periodic acid-Schiff, and examined by light microscopy. The following variables were measured in the intestine: villus height, villus width, depth of crypts, goblet cell number and goblet cell diameter in each segment, performed with a light microscope using EPIX XCAP software (Standard version, Olympus, Waltham, MA, USA). Four segments for each biological sample and five biological samples per treatment group were used. Villi height was measured using the lamina propria as the base; villi width, depth of the crypt and the number of goblet cell were measured per side of a longitudinal section through the villus; goblet cell size was measured as the diameter of the goblet cells (µm^2^). Villi surface area was calculated from the villus height and width at half height as according to Uni et al. [42]. For the Alcian Blue and Periodic acid-Schiff stain, the segments were only counted for the types of goblet cells in the villi epithelium, goblet cells within the crypts and the mucus layer thickness. Goblet cells were enumerated on 10 villi/sample, and the means were utilized for statistical analysis. The liver was stained with hematoxylin-esoin (H&E) for standard microscopy and visualized using the same light microscope. Mean adipocyte diameter was determined by random, utilizing the EPIX XCAP software (standard version, Olympus, Waltham, MA, USA), by enumerating 10 adipocytes/segment/sample, and the means were utilized for statistical analysis. 

### 2.15. Statistical Analysis

All values are expressed as the means and standard deviations. Experimental treatments for the in ovo assay were arranged in a completely randomized design. The results were analyzed by ANOVA. For significant “*p*-value”, post hoc Duncan test was used to compare test groups. Statistical analysis was carried out using SPSS version 20.0 software (IBM, Armonk, USA). The level of significance was established at *p* < 0.05.

## 3. Results

### 3.1. Concentration of Iron, Zinc, Phytic Acid and Dietary Fiber and the Phytate:Iron Ratio in Chia Flour and in Chia Extract

The iron and zinc concentrations, insoluble fiber content, phytic acid and the phytate:iron ratio were higher (*p* < 0.05) in the chia seed compared to the chia extract (Table 2). However, the content of soluble fiber was significantly greater (*p* < 0.05) in the chia extract relative to chia seed. 

### 3.2. Polyphenol Profile in Chia Flour

The concentration of the five most prevalent polyphenolic compounds found in chia is presented in Table 3. Chia presented high concentrations of rosmarinic acid and rosmarinyl glucoside. In addition, we observed the presence of ferulic acid, caffeic acid and protocatechuic acid.

### 3.3. In Ovo Assay (Gallus Gallus Model)

#### 3.3.1. Hb Concentration

The Hb values were significantly (*p* < 0.05) higher in the “2.5% chia” extract treatment group compared to the 18 Ω H_2_O and non-inject group. The other treatments did not differ from each other (Table 4).

#### 3.3.2. Iron and Zinc Concentration in Liver and Serum

As shown in Table 5, there were no significant (*p* > 0.05) differences in liver iron concentration and serum zinc concentration between treatment groups. However, “1% chia” extract treatment increased (*p* < 0.05) the zinc liver content compared to non-inject treatment. In addition, we observed that “5% chia” extract treatment showed a lower (*p* < 0.05) serum iron concentration when compared to the 18 Ω H_2_O and inulin groups. In general, different concentrations of chia extract did not affect iron and zinc concentrations in liver and serum. 

#### 3.3.3. Gene Expression of Fe- and Zn-Related Genes

**Figure 1:**
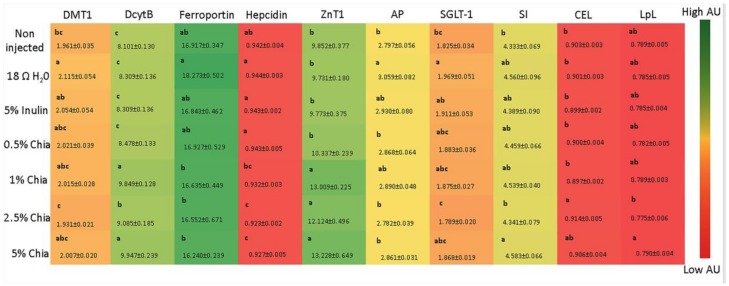
Effect of the intra-amniotic administration of experimental solutions on intestinal and liver gene expression. Values are the means ± SEM, *n* = 10. ^a–c^ Per gene, treatments groups not indicated by the same letter are significantly different (*p* < 0.05). DMT1, Divalent metal transporter 1; Dcytb, Duodenal cytochrome b; ZnT1, Zinc transporter 1; AP, Amino peptidase; SGLT1, Sodium-Glucose transport protein 1; SI, Sucrose isomaltase; CEL, Carboxyl ester lipase; LpL, Lipoprotein lipase.

The gene expression of DMT1 was lower (*p* < 0.05) in the group treated with 2.5% chia soluble extract compared to the inulin and 18 Ω H_2_O groups (Figure 1). However, other various concentrations of chia soluble extract did not affect the expression of DMT1 (*p* > 0.05). The relative expression of DcytB and hepcidin was significantly up-regulated (*p* < 0.05) in the 1%, 2.5% and 5% chia extract. The groups treated with 1%, 2.5% and 5% chia extract showed lower (*p* > 0.05) ferroportin gene expression compared to the 18 Ω H_2_O injected group. However, no differences (*p* > 0.05) were observed between chia treatment groups. The relative expression of ZnT1 was significantly up-regulated (*p* < 0.05) in the 1%, 2.5% and 5% chia extract. 

#### 3.3.4. Gene Expression of BBM Functional Proteins

The gene expression of aminopeptidase (AP), sodium-glucose transport protein 1 (SGLT1) and sucrase isomaltase (SI) are used as biomarkers of brush border membrane digestive and absorptive functions. AP and SGLT1 gene expression did not differ (*p* > 0.05) between groups treated with chia extract. However, the gene expression of SI was higher (*p* < 0.05) in “5% chia” extract treatment group compared to the “2.5% chia” extract treatment group (Figure 1). 

#### 3.3.5. Gene Expression of Lipids Metabolism Protein

The gene expressions of carboxyl ester lipase (CEL) and lipoprotein lipase (LpL) are used as biomarkers of lipid metabolism. As shown in Figure 1, the “2.5% chia” extract treatment group presented higher (*p* > 0.05) CEL expression compared to the control groups. However, the gene expression of LpL did not differ between chia extract groups and control groups (*p* < 0.05).

#### 3.3.6. Cecum-to-Body-Weight Ratio

**Figure 2:**
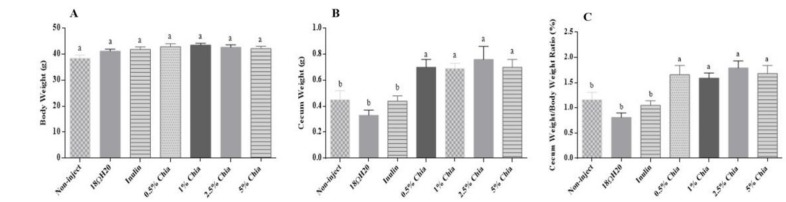
The effect of chia on the: (**A**) body weight; (**B**) cecum weight; and (**C**) cecum weight/body weight ratio (%). Values are the means ± SEM *n* = 15. ^a,b^ Within a column, means without a common letter are significantly different (*p* < 0.05).

As shown in Figure 2, the chia soluble extract treatment groups showed a higher (*p* < 0.05) cecum weight (B) and cecum weight/body weight ratio (C) compared to control groups (*p* < 0.05). However, no significant difference (*p* > 0.05) was observed in body weight (A) among treatment groups and controls. 

#### 3.3.7. Microbial Analysis

**Figure 3:**
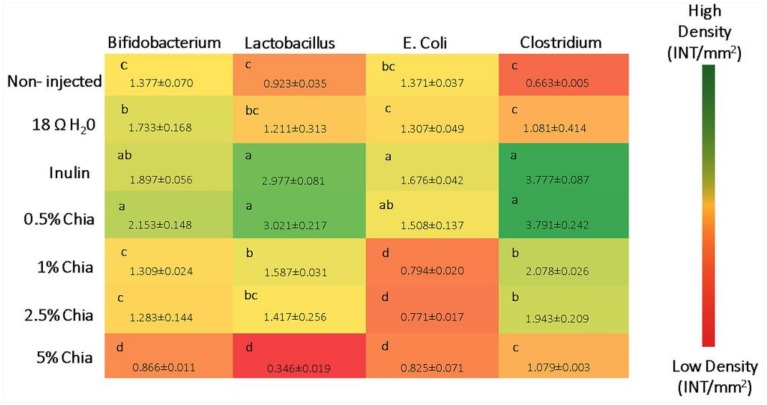
Genera- and species-level bacterial populations (AU) from cecal contents measured on the day of hatch. Values are the means ± SEM, *n* = 10. ^a–d^ Per bacterial category, treatment groups not indicated by the same letter are significantly different.

As shown in Figure 3, the relative abundance of both *Bifidobacterium* and *Lactobacillus* genera, increased (*p* < 0.05) in the “0.5% chia” treatment, relative to the 18 Ω H_2_O group and non-injected group. The “5% chia” treatment group showed a lower (*p* < 0.05) concentration of these bacterial populations compared to the other groups. The relative abundance of *E. coli* significantly decreased (*p* < 0.05) in the 1%, 2.5% and 5% chia extract treatment groups compared to the control groups. The relative abundance of *Clostridium* was significantly (*p* < 0.05) lower in the non-inject group, 18 Ω H_2_O group and “5% chia” treatment group. These results suggest that a lower concentration of chia extract may positively affect gut health.

#### 3.3.8. Glycogen Analysis

No significant difference was observed in pectoral muscle glycogen content between groups (Table 6, *p* > 0.05). 

#### 3.3.9. Morphometric Data for Villi, Depth of Crypts and Goblet Cell

**Figure 4:**
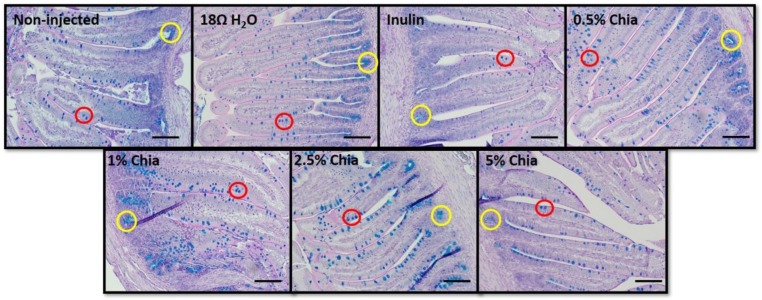
Representations of the intestinal morphology of each treatment group are shown (Alcian Blue and Periodic acid-Schiff Stain). The yellow circles indicate crypts within the villi and the red circles indicate goblet cells on the villi. Bar = 50 µm.

**Figure 5:**
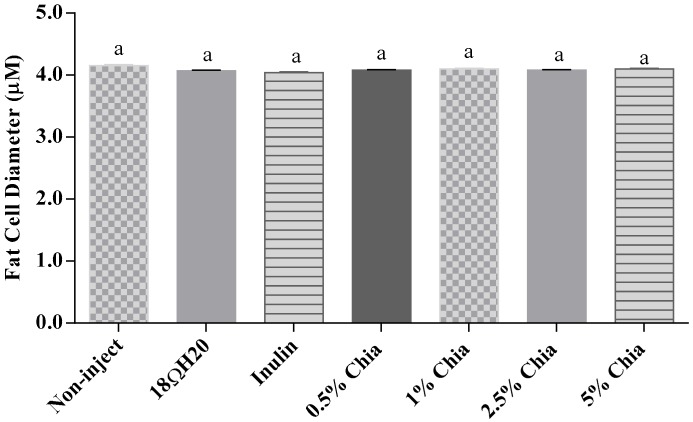
Fat cell diameter. Values are the means ± SEM, *n* = 5. ^a^ Treatment groups not indicated by the same letter are significantly different.

The villus surface areas, villi length, width and the number of goblet cells were significantly (*p* < 0.05) higher in all chia extract treatment groups compare to controls (Table 7 and Table 8), indicating that soluble extracts from chia had a positive effect on intestinal development, through the proliferation of enterocytes, and the increased number in mucus-producing cells. However, there were no significant (*p* > 0.05) differences in crypt depth and mucus layer width between treatment groups. Further, all chia extract treatments increased (*p* < 0.05) the diameter of goblet cells compared to controls. In relation to the types of goblet cells observed (acidic, neutral, mixed), we can note that the administration of 2.5% chia soluble extracts reduced (*p* < 0.05) the number of neutrals goblet cells compared to the control groups. In addition, the administration of 2.5% and 5% chia soluble extracts increased (*p* < 0.05) the number of acidic goblet cells, whereas the administration of 1% and 2.5% chia extract caused an increase (*p* < 0.05) in mixed goblet cells, compared to controls. In relation to the types of goblet cells in the crypt epithelium, the administration of 0.5% chia soluble extract increased (*p* < 0.05) the number of neutrals goblet cells compared to controls. In addition, the administration of 2.5% chia extracts increased (*p* < 0.05) the number of mixed goblet cells compared to controls. The number of acid goblet cells did not differ (*p* > 0.05) between groups (Figure 4). No significant differences between treatment groups were measured in fat cell diameter (*p* > 0.05, Figure 5).

#### 3.3.10. Hepatic Morphometric Measurement

As shown in Figure 4, no significant differences were observed in hepatic fat cell diameter between all treatment groups (*p* > 0.05). 

## 4. Discussion

Chia is a good source of dietary fiber, which was demonstrated to have a beneficial effect on intestinal health [29]. However, until now, the potential effects of soluble extracts from chia seed on the intestinal microbiota, intestinal morphology and mineral bioavailability, such as iron and zinc, were not investigated. Further, it is important to highlight that the alterations in microbiota populations, due the consumption of dietary fiber, may be associated, directly or indirectly, to the increased dietary bioavailability of iron and zinc in vulnerable populations [13,15,18,27]. The present study indicates that the in ovo administration of soluble extracts from chia seed increased the intestinal villus surface area, villi length, villi width, goblet cell number and goblet cell size (diameter), as well as cecum weight (used as biomarker of microbial presence and activity). In addition, the administration of chia seed soluble extracts up-regulated the expression of proteins related to zinc metabolism. Further, the chia soluble extract (0.5%) increased the *Bifidobacterium* and *Lactobacillus* relative abundance in cecum content. 

According to our results, the hemoglobin concentration results corroborate with our findings of serum iron. We did not observe a change in liver iron concentrations, due to the short time of exposure of the soluble extracts, which was not sufficient to cause a modification in hepatic iron storage. This was in agreement with previous observations that evaluated the effects of intra-amniotic raffinose and stachyose administration on Fe status, as the results showed no significant differences in hemoglobin values between treatment groups [10]. Further, another study that assessed the effect of the intra-amniotic administration of bean soluble extracts on iron status indicated that bean extracts did not affect serum or liver iron concentrations [12]. A similar result was observed post intra-amniotic administration of wheat extracts [14]. In addition, a BBM Fe metabolism-related gene expression analysis of DcytB, DMT, ferroportin and hepcidin was conducted. DcytB is the protein responsible for reducing Fe^3+^ to Fe^2+^ in the apical membrane of the enterocyte [10,43]. DMT1 plays a key role in Fe^2+^ transport into the enterocyte, being considered the major Fe intestinal transporter [10,43], whereas ferroportin is the protein that transports Fe^2+^ from the enterocyte into the bloodstream [10,43]. In the current study, the administration of 1%, 2.5% and 5% chia soluble extract solutions up-regulated the expression of DcytB, which in return may increase the transportation of Fe by DMT1 into the enterocyte, and as previously demonstrated, this effect can potentially increase iron absorption efficiency in a long-term feed trial [12]. Further, we investigated hepcidin gene expression as the key iron-regulatory hormone that controls systemic iron homeostasis, as hepcidin is able to down regulate the expression of ferroportin [44,45]. Further, the increase in hepcidin production is stimulated by iron loading and inflammation [46,47]. In the present study, hepcidin gene expression was lower (*p* < 0.05) in the 1%, 2.5% and 5% chia soluble extract groups compared to the inulin and water groups, which suggests that in a long-term feeding trial, the dietary inclusion of chia may have a positive effect on Fe-related proteins.

ZnT1 is the only transporter of the ZnT transporters family that is localized on the enterocyte’s basolateral membrane and functions by exporting cytosolic zinc into the extracellular space [48], an up-regulation in ZnT1 mRNA gene expression may occur under increased cellular zinc levels [49]. In the current study, the groups treated with chia seed soluble extract (1%, 2.5% and 5%) shown a gene expression up-regulation (*p* < 0.05) of ZnT1 compared to the other groups, although the zinc serum concentrations did not differ between experimental groups. 

Previous studies demonstrated the potential beneficial effects of soluble extract from various sources and plant origin compounds (such as raffinose, stachyose, diadzein, bean, and wheat) on BBM functionality and intestinal bacterial populations [10,11,12,13,27]. In the current study, the expression of BBM functional genes (AP, SI and SGLT1) was not affected by the chia seed soluble extract administration, due to the short exposure time. However, in relation to microbial populations, there was an increasing abundance of *Lactobacillus* (*p* < 0.05), and *Bifidobacterium* (*p* < 0.05) in the cecal contents of animals received 0.5% chia soluble extracts compared to the 18 Ω H_2_O and non-injected group. Further, we observed an increased abundance in *Lactobacillus* (*p* < 0.05), *Bifidobacterium* (*p* < 0.05), *E. Coli* (*p* < 0.05) and *Clostridium* (*p* < 0.05) in the cecal contents of the animals that received 0.5% chia seed soluble extracts compared to other groups treated with chia seed extract. It is important to highlight that the increase in *Lactobacillus* and *Bifidobacterium* abundance, due the consumption of dietary fiber, may further contribute, directly or indirectly, to the increased bioavailability of iron and zinc in vulnerable populations, as these bacterial genera produce short-chain fatty acids (SCFAs), which reduce the intestinal pH, and therefore, may increase mineral (as Fe and Zn) solubility and therefore absorption [50]. *Bifidobacterium* and *Lactobacillus* can break down non-digestible fiber (prebiotics), due to their 1,2-glycosidase activity, leading to greater SCFA production [16,27,39], culminating with the increase in the absorption of iron and zinc. 

The morphological parameters described in the current study, including villi development parameters and the crypt depth, are used as indicators of intestinal health, functionality and development [51]. The administration of chia seed soluble extracts, regardless of the concentration used, increased all parameters related to intestinal villi. These values (villus surface area, villus length and width) were significantly higher (*p* < 0.05) in the 5.0% chia group and relative to all other groups. This can be explained by the potential increased proliferation of intestinal cells in the short term, due the presence of soluble fiber, leading to hyperplasia and/or hypertrophy of intestinal cells and potentially enhancing the absorptive and digestive capacity of the villi BBM [29]. Another explanation is that the tested extracts had potentially increased butyrate production, which may lead to enterocyte proliferation [52]. Added to these factors, the soluble extract of chia seed contains a high concentration of phenolic compounds, among them are rosmarinic acid and rosmarinyl glucoside, which present the ability to affect intestinal morphology [53], increasing the villus height, crypt depth ratio, and muscularis thickness, as observed in the study that evaluated the administration of dietary polyphenol concentrate previously performed in *Gallus gallus* [54]. The morphological results agree with our cecum weight and cecum weight/body weight ratio observations. All experimental groups showed a higher (*p* > 0.05) cecal weight (Figure 2B) post intra-amniotic soluble extract administration, indicating, and as previously suggested, increased cecal bacterial populations activity [10,12,13]. As for crypt depth, no differences between the experimental groups were observed, since duodenal crypts require a longer time to allow cellular proliferation. However, the intestinal crypts are meager and are able to rise to the surface of the villus, increasing the number of enterocytes in intestinal villi [52]—a phenomenon that was observed in the current study. Additionally, we observed increased goblet cell number and goblet cell diameter, which suggests an increased production of mucus that coats the intestinal lumen. As previously suggested, this may increase the intestinal BBM digestive and absorptive capabilities, and may indirectly increase the bioavailability of dietary components as suggested by the effects observed on the morphometric parameters [55,56,57]. The increase in “acidic goblet cells”, containing acidic mucin due to the administration of 2.5% and 5% chia soluble extracts, may contribute to the reduction of intestinal pH, which in the long term, may lead to increased solubilization and uptake of iron and zinc and affect intestinal microbial profile [14,39]. The increase in “acidic goblet cells” was previously observed in a study that evaluated the effects of the intra amniotic administration of carbohydrate solution (containing maltose, sucrose and dextrin) on mucin content, goblet cell development, and levels of mucin mRNA in the *Gallus gallus* small intestine [58].

In general, previous studies showed a positive effect of prebiotic administration on intestinal morphology [10,13,25,51,52], for example, the intra-amniotic administration of raffinose and stachyose increased villus surface area compared to the control [10]. Similar results were observed by Hou et al. [13], who evaluated the effect of chickpea and lentil prebiotics administration in ovo. In another study, the authors evaluated the development of morphological parameters in *Gallus gallus*, and the results showed that the administration of a synthetic prebiotic increased the villus width and crypt depth. The prebiotic had no impact on villus height, villus surface area, and muscular thickness compared to the animals that received saline solution administration [51]. Bogucka et al. [52] evaluated the effect of inulin administration on the development of the intestinal villi and the number of goblet cells in the small intestine on the 1st and the 4th day post hatch (*Gallus gallus*) and the study indicated that on day one, the villus height did not differ among experimental groups. However, the villus width, villus surface area and crypt depth were lower in the prebiotic group. On day four, the inulin group showed a lower villus width, villus surface area and crypt depth [52]. Another study that evaluated the effect of the intra aminiotic administration of wheat bran prebiotic extract indicated increased villus height, goblet cell diameter and number in all treatment groups [11]. Further, Mista et al. [25] evaluated the effect of intra amniotic administered prebiotics on the development of the small intestine (*Gallus gallus*) and found that prebiotics did not affect the villus length, but did increase the crypt depth. 

The observations described in the current study suggest that dietary chia seed consumption may be an effective strategy to reduce dietary iron and zinc deficiency and to potentially improve intestinal health. Overall, the up-regulation of Zn gene expression and the DcytB-Fe metabolism protein, the increase in villus surface area, villus length, villus width, goblet cell number and goblet cell diameter as well as cecum weight suggest that chia is a promising food ingredient that may improve mineral bioavailability and intestinal morphology. Hence, long-term feeding trials assessing the dietary effects of chia are now warranted. 

## 5. Conclusions

The intra-amniotic (in ovo) administration of chia seed soluble extracts with putative prebiotic effects improved the intestinal morphology and up-regulated Zn-related protein gene expression. Further, chia seed soluble extract administration affected the intestinal microbiota and iron-related gene expression. The current study is the first to investigate the effects of chia seed soluble extracts with a potential prebiotic effect in vivo; thus, future studies aimed to assess dietary chia seed in a long-term feeding trials should be conducted, since chia may be a viable dietary ingredient that may improve intestinal health and contribute to intestinal mineral absorption.

## Figures and Tables

**Table 1:** The sequences of the primers used in this study.

Analyte	Forward Primer (5′–3′)	Reverse Primer (5′–3′)	Base Pair	GI Identifier
DMT1	TTGATTCAGAGCCTCCCATTAG	GCGAGGAGTAGGCTTGTATTT	101	206597489
Ferroportin	CTCAGCAATCACTGGCATCA	ACTGGGCAACTCCAGAAATAAG	98	61098365
Dcytb	CATGTGCATTCTCTTCCAAAGTC	CTCCTTGGTGACCGCATTAT	103	20380692
Hepcidin *	GAGCAAGCCATGTCAAGATTTC	GTCTGGGCCAAGTCTGTTATAG	132	8056490
ZnT1	GGTAACAGAGCTGCCTTAACT	GGTAACAGAGCTGCCTTAACT	105	54109718
SI	CCAGCAATGCCAGCATATTG	CGGTTTCTCCTTACCACTTCTT	95	2246388
AP	CGTCAGCCAGTTTGACTATGTA	CTCTCAAAGAAGCTGAGGATGG	138	45382360
SGLT1	GCATCCTTACTCTGTGGTACTG	TATCCGCACATCACACATCC	106	8346783
LPL *	TGCTCAGATGCCCTACAAAG	TCTCGTCTAGAGTGCCATACA	119	396219
CEL *	ATGCTGCTGACATCGACTAC	TTCTGAAGTGGACGGTTGATAG	97	417165
18S rRNA *	GCAAGACGAACTAAAGCGAAAG	TCGGAACTACGACGGTATCT	100	7262899

DMT1, Divalent metal transporter 1; Dcytb, Duodenal cytochrome b; Znt 1, Zinc transporter 1; SI, Sucrose isomaltase; AP, Amino peptidase; SGLT1, Sodium-Glucose transport protein 1; LPL, Lipoprotein lipase; CEL, Carboxyl ester lipase; 18S rRNA, 18S Ribosomal subunit. * liver analyses.

**Table 2:** Concentration of iron, zinc, dietary fiber and phytic acid in chia flour and in chia extract.

Treatment Group	Iron (µg/g)	Zinc (µg/g)	Insoluble Fiber (g/100g)	Soluble Fiber (g/100g)	Phytic Acid (g/100g)	Phytic Acid: Iron Ratio
Chia seed	110.25 ± 4.97 ^a^	57.82 ± 0.40 ^a^	34.67 ± 1.84 ^a^	4.01 ± 0.21 ^b^	0.71 ± 0.02 ^a^	5.47 ^a^
Chia extract	41.46 ± 0.89 ^b^	31.29 ± 0.89 ^b^	23.53 ± 1.74 ^b^	19.68 ± 0.76 ^a^	0.08 ± 0.00 ^b^	1.60 ^b^

Values are the means ± SEM, *n* = 5. ^a,b^ Treatment groups not indicated by the same letter are significantly different (*p* < 0.05).

**Table 3:** Polyphenol profile present in chia flour.

Polyphenolic Compounds	Mean Peak Area (mAU-min/10^6^)
Rosmarinic acid	42.30 ± 1.90
Rosmarinyl glucoside	57.70 ± 0.02
Ferulic acid	1.19 ± 0.06
Caffeic acid	0.76 ± 0.38
Protocatechuic acid	0.21 ± 0.03

Values are the means ± SEM, *n* = 10. mAU: milli absorbance unit; min: minutes.

**Table 4:** Blood hemoglobin (Hb) concentrations (g/dL).

Treatment Group	Hb (g/dL)
Non-injected	5.93 ± 0.00 ^b^
18 Ω H_2_O	5.52 ± 1.49 ^b^
Inulin	7.76 ± 1.16 ^a,b^
0.5% Chia	7.08 ± 1.16 ^a,b^
1.0% Chia	9.51 ± 1.34 ^a,b^
2.5% Chia	10.41 ± 1.37 ^a^
5.0% Chia	10.06 ± 2.48 ^a,b^

Values are the means ± SEM, *n* = 10. ^a,b^ Treatment groups not indicated by the same letter are significantly different (*p* < 0.05).

**Table 5:** Iron and zinc concentrations (ppm).

Treatment Group	Liver		Serum	
	Iron (µg/g)	Zinc (µg/g)	Iron (µg/g)	Zinc (µg/g)
Non-injected	35.28 ± 2.52 ^a^	14.77 ± 1.26 ^b^	3.14 ± 0.25 ^a,b,c^	0.001 ± 0.000 ^a^
18 Ω H_2_O	41.00 ± 3.24 ^a^	16.10 ± 1.57 ^a,b^	4.04 ± 0.52 ^a,b^	0.002 ± 0.000 ^a^
Inulin	40.92 ± 3.32 ^a^	16.39 ± 2.43 ^a,b^	4.24 ± 0.96 ^a^	0.001 ± 0.000 ^a^
0.5% Chia	35.57 ± 3.16 ^a^	18.45 ± 1.13 ^a,b^	2.99 ± 0.44 ^a,b,c^	0.002 ± 0.000 ^a^
1.0% Chia	43.17 ± 4.08 ^a^	21.63 ± 2.59 ^a^	2.36 ± 0.24 ^a,b^	0.003 ± 0.001 ^a^
2.5% Chia	33.52 ± 1.67 ^a^	16.60 ± 1.41 ^a,b^	3.23 ± 0.63 ^a,b,c^	0.001 ± 0.000 ^a^
5.0% Chia	35.88 ± 2.81 ^a^	17.87 ± 2.52 ^a,b^	1.59 ± 0.29 ^c^	0.002 ± 0.000 ^a^

Values are the means ± SEM, *n* = 10. ^a,b,c^ Treatment groups not indicated by the same letter are significantly different (*p* < 0.05).

**Table 6:** Concentration of glycogen in pectoral muscle.

Treatment Group	Glycogen Concentration (mg/g)
Non-injected	0.17 ± 0.04 ^a^
18 Ω H_2_O	0.21 ± 0.05 ^a^
Inulin	0.29 ± 0.06 ^a^
0.5% Chia	0.13 ± 0.03 ^a^
1.0% Chia	0.31 ± 0.06 ^a^
2.5% Chia	0.26 ± 0.08 ^a^
5.0% Chia	0.29 ± 0.15 ^a^

Values are the means ± SEM, *n* = 10. ^a^ Treatment groups not indicated by the same letter are significantly different (*p* < 0.05).

**Table 7:** Effect of the intra-amniotic administration of experimental solutions on the duodenal small intestinal villus and crypt.

Treatment Group	Villus Surface Area (mm^2^)	Villus Length (µM)	Villus Width (µM)	Depth of Crypts (µM)	Mucus Layer Width (µM)
Non-injected	170.29 ± 5.33 ^c^	248.64 ± 2.83 ^c^	43.26 ± 0.42 ^c^	12.76 ± 0.10 ^a^	2.21 ± 0.27 ^a^
18 Ω H_2_O	127.13 ± 8.16 ^c^	204.30 ± 3.40 ^d^	39.24 ± 0.37 ^d^	12.60 ± 0.09 ^a^	2.32 ± 0.15 ^a^
Inulin	130.00 ± 9.42 ^c^	208.90 ± 3.63 ^d^	41.20 ± 0.56 ^c,d^	13.01 ± 0.10 ^a^	2.36 ± 0.1 ^a^
0.5% Chia	237.53 ± 7.98 ^b^	323.85 ± 3.51 ^b^	46.42 ± 0.40 ^b^	12.49 ± 0.08 ^a^	2.41 ± 0.25 ^a^
1.0% Chia	234.78 ± 7.36 ^b^	298.82 ± 2.43 ^b^	49.70 ± 0.51 ^b^	13.08 ± 0.09 ^a^	2.22 ± 0.10 ^a^
2.5% Chia	264.95 ± 2.74 ^b^	334.44 ± 5.62 ^b^	50.15 ± 0.57 ^b^	12.83 ± 0.10 ^a^	2.20 ± 0.13 ^a^
5.0% Chia	343.93 ± 9.38 ^a^	374.47 ± 5.50 ^a^	58.18 ± 0.59 ^a^	12.71 ± 0.11 ^a^	2.15 ± 0.14 ^a^

Values are the means ± SEM, *n* = 5. ^a–d^ Treatment groups not indicated by the same letter are significantly different (*p* < 0.05).

**Table 8:** Effect of the intra-amniotic administration of experimental solutions on the goblet cells.

Treatment Group	Goblet Cell Diameter (µM)	Total Goblet Cell Number (Unit)	Villus Goblet Cell Number (Unit)			Crypts Goblet Cell Number (Unit)		
			Neutral	Acid	Mixed	Neutral	Acid	Mixed
Non-injected	4.20 ± 0.03 ^c^	21.23 ± 0.24 ^c^	2.50 ± 0.33 ^a,b^	8.77 ± 0.23 ^b^	9.11 ± 0.33 ^c^	0.01 ± 0.00 ^b^	10.36 ± 0.57 ^a^	0.47 ± 0.15 ^c^
18 Ω H_2_O	4.10 ± 0.03 ^c^	20.18 ± 0.26 ^c^	2.14 ± 0.24 ^b^	8.05 ± 0.74 ^c^	9.62 ± 0.47 ^c^	0.10 ± 0.00 ^b^	9.69 ± 0.55 ^a^	1.64 ± 0.16 ^a^
Inulin	4.89 ± 0.06 ^b^	24.88 ± 0.20 ^b^	3.90 ± 0.99 ^a^	8.67 ± 0.48 ^b^	11.02 ± 1.02 ^b,c^	0.01 ± 0.00 ^b^	10.32 ± 0.36 ^a^	0.43 ± 0.14 ^c^
0.5% Chia	5.48 ± 0.03 ^a,b^	28.59 ± 0.32 ^a^	3.63 ± 0.25 ^a,b^	10.76 ± 0.71 ^a,b^	14.26 ± 0.51 ^a^	0.46 ± 0.12 ^a^	10.02 ± 0.91 ^a^	0.89 ± 0.06 ^b,c^
1.0% Chia	5.36 ± 0.05 ^a,b^	29.19 ± 0.29 ^a^	2.07 ± 0.17 ^b^	11.43 ± 0.61 ^a,b^	15.55 ± 0.71 ^a^	0.09 ± 0.05 ^b^	9.65 ± 1.05 ^a^	1.30 ± 0.11 ^a,b^
2.5% Chia	5.61 ± 0.02 ^a^	29.61 ± 0.40 ^a^	1.63 ± 0.16 ^c^	13.70 ± 1.53 ^a^	13.72 ± 1.35 ^a^	0.05 ± 0.02 ^b^	9.45 ± 0.49 ^a^	1.45 ± 0.30 ^a^
5.0% Chia	5.42 ± 0.06 ^a,b^	29.91 ± 0.39 ^a^	2.55 ± 0.43 ^a,b^	13.13 ± 1.35 ^a^	13.20 ± 1.51 ^a,b^	0.18 ± 0.06 ^a,b^	9.88 ± 0.13 ^a^	0.82 ± 0.21 ^b,c^

Values are the means ± SEM, *n* = 5. ^a–c^ Treatment groups not indicated by the same letter are significantly different (*p* < 0.05).
